# Neglected epiphyseal injuries of the distal end of the radius with ulnar impaction: analysis of distal osteotomy of both bones using a dorsal midline approach

**DOI:** 10.1007/s10195-016-0423-x

**Published:** 2016-07-28

**Authors:** Paritosh Gogna, Sahil Gaba, Reetadyuti Mukhopadhyay, Rajesh Rohilla, Amanpreet Singh

**Affiliations:** 1 Department of Orthopaedics and Rehabilitation, Pt B.D. Sharma Post Graduate Institute of Medical Sciences, Rohtak, Haryana India; 2 Department of Orthopaedics, All India Institute of Medical Sciences (AIIMS), New Delhi, India

**Keywords:** Neglected epiphyseal injury of distal-end radius, Osteotomy, Ulnar impaction

## Abstract

**Background:**

To evaluate results of a technique for treating neglected epiphyseal injuries of the distal radius with ulnar impaction.

**Materials and methods:**

This retrospective study involved six cases (four males; two females), all of whom sustained the primary injury during childhood (range 9–12 years of age). All presented with wrist deformity and ulnar-sided wrist pain. They were managed with osteotomy of the distal radius, osteotomy and shortening of the ulna, harvesting the bone grafts, and distal radioulnar joint (DRUJ) reduction performed simultaneously through a dorsal midline approach. Mean follow-up was 30 months (range 24–36).

**Results:**

Deformity correction and pain relief was observed in all patients. Flexion arc increased from an average of 60° to 102.5°, supination from an average of 31.67° to 67.50°, and pronation from an average of 30.83° to 61.67°. The mean preoperative DASH score was 87.5, which improved to 18.72 postoperatively.

**Conclusion:**

Neglected epiphyseal injuries of the distal radius are difficult to manage and many variations are described for handing each of the associated problems. Our technique provides an option for managing this injury with an easy surgical approach, single incision, and cost effectiveness. All the four components of the surgery, which include osteotomy of the distal radius, osteotomy of the ulna, harvesting the bone grafts, and DRUJ reduction were done through a single incision and in a single sitting.

*Level of evidence* IV.

## Introduction

Malunion is the commonest deformity in adult distal radius fractures, which complicates ~23 % of non-surgically treated, and 11 % of operatively treated fractures [1–4]. The incidence in children is much lower, as any malunion of the distal end of the radius in children usually remodels itself [5, 6]. However, this may not always occur when there is associated damage to the physeal plate, leading to partial or complete growth arrest [7]. Other factors which affect remodeling are age of the patient at the time of fracture, the distance between the fracture and the epiphyseal plate, and the extent of residual angulation following reduction [5–7]. An anatomically reduced distal radius can also lead to deformity later on due to damage to the physis [8]. Multiple attempts at reduction and late re-manipulation at more than 7 days post injury are known risk factors for physeal arrest [7, 9, 10]. The incidence of physeal closure is 7–10 % according to Lee [10]. Malunions may manifest themselves variedly, ranging from asymptomatic radiographic abnormalities to disabling deformities associated with significant pain and functional impairment [4, 11]. Treatment in the form of corrective osteotomy was first proposed by Meyerding and Overton in 1935 [12]. Since then, many techniques have been described which have their own pros and cons. Most research in this regard has been done in adult patients.

Neglected injuries in skeletally immature patients pose unique challenges, especially those with associated physeal arrest. These injuries do not remodel completely, and the normal ulnar growth later leads to DRUJ dislocation and ulnar impaction. If recognized early, physeal bar excision and fat interposition, along with distal ulnar epiphysiodesis can be done [13]. However, once ulnar abutment or impingement is present and potential for growth is over, a more invasive procedure is usually required. Neglected distal radius malunion with positive ulnar variance and distal radioulnar joint (DRUJ) disruption is a challenging situation for the surgeon. We describe our results with a simple technique in which all four components of the surgery, which include osteotomy of the distal radius, osteotomy of the ulna, harvesting the bone grafts,and DRUJ reduction can be done through a single incision and in a single sitting.

## Materials and methods

**Fig. 1:**
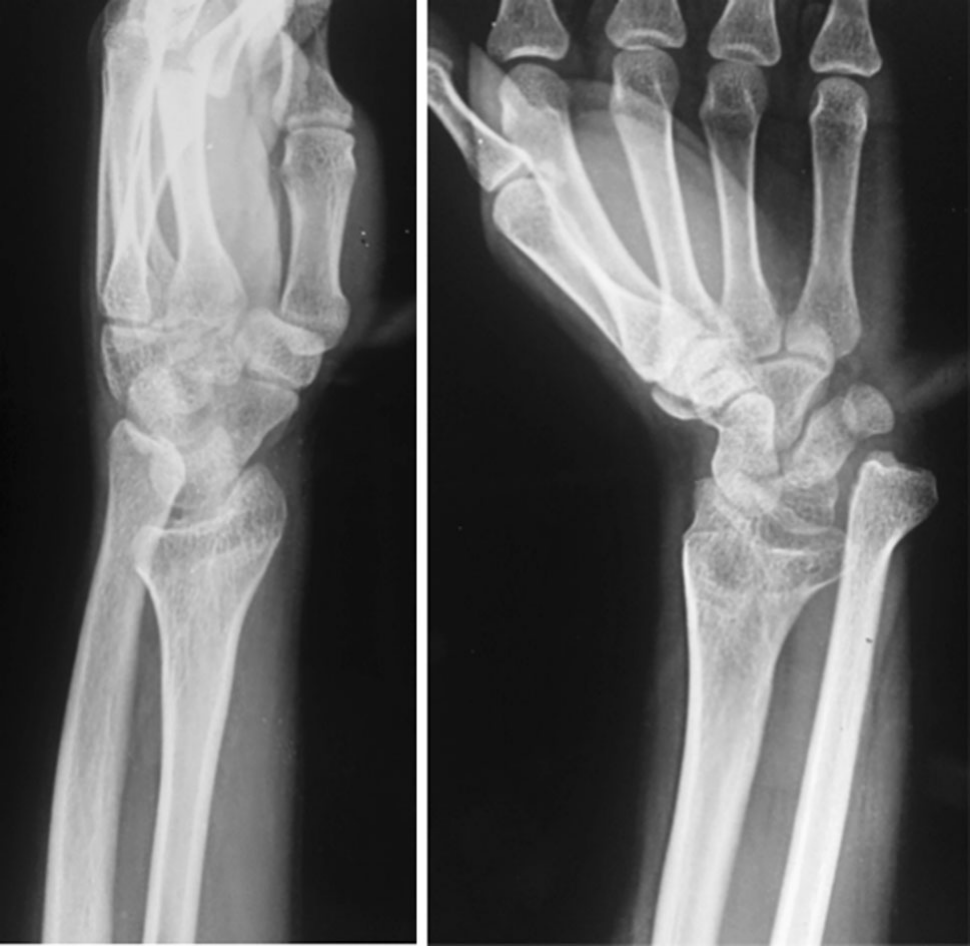
Preoperative radiographs of a 21-year-old male with neglected epiphyseal injury of the distal end of the radius with DRUJ disruption and dorsal ulnar subluxation

This retrospective study involved six patients (four males and two females) who presented to us with the chief complaints of unsightly deformity of the wrist (radial deviation and extension deformity) with ulnar-sided wrist pain and a history of injury to the wrist during childhood (range 9–12 years; mean 10.66 years). The mechanism of injury was a fall on an outstretched hand in four cases, while in two cases it was unknown. Four cases involved the dominant side, two the non-dominant side. None of these were managed by a trained surgeon at the time of initial injury, and no patient had previous medical records or radiographs. On examination there was restriction of flexion at the wrist (average 29.16°), and rotations (supination 31.66° and pronation 30.83°). The piano key sign (ulnar ballottement) was positive in all cases. PA and lateral radiographs of the wrist were performed (Fig. 1), and based on it we made the diagnosis of neglected epiphyseal injury of the distal end of the radius with DRUJ disruption and ulnar impaction. Contralateral normal wrist radiographs were also obtained to aid in pre-operative planning and to ascertain the degree of angular correction needed at the osteotomy site in both sagittal (volar/dorsal tilt) and coronal (radial inclination) planes. Our diagnosis was based on history and radiological criteria of malunion as described by Graham [14]. Patients were taken up for surgery after written informed consent.

**Fig. 2:**
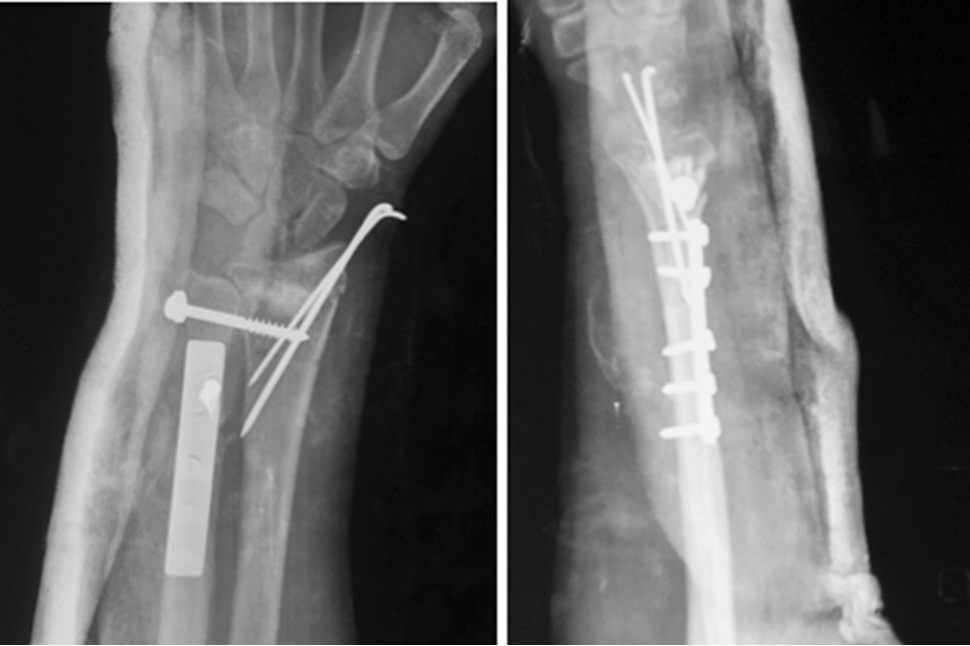
Post operative radiographs of the same patient, showing restoration of distal radius anatomy

**Fig. 3:**
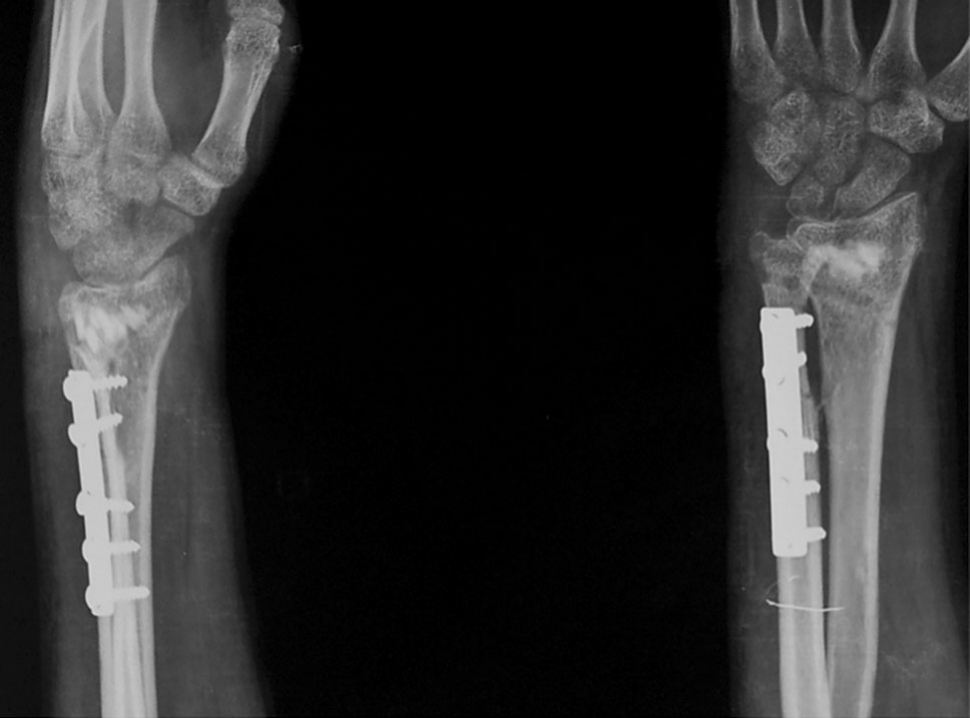
Radiographs 6 weeks after the surgery, after removal of K wires and screw

**Fig. 4:**
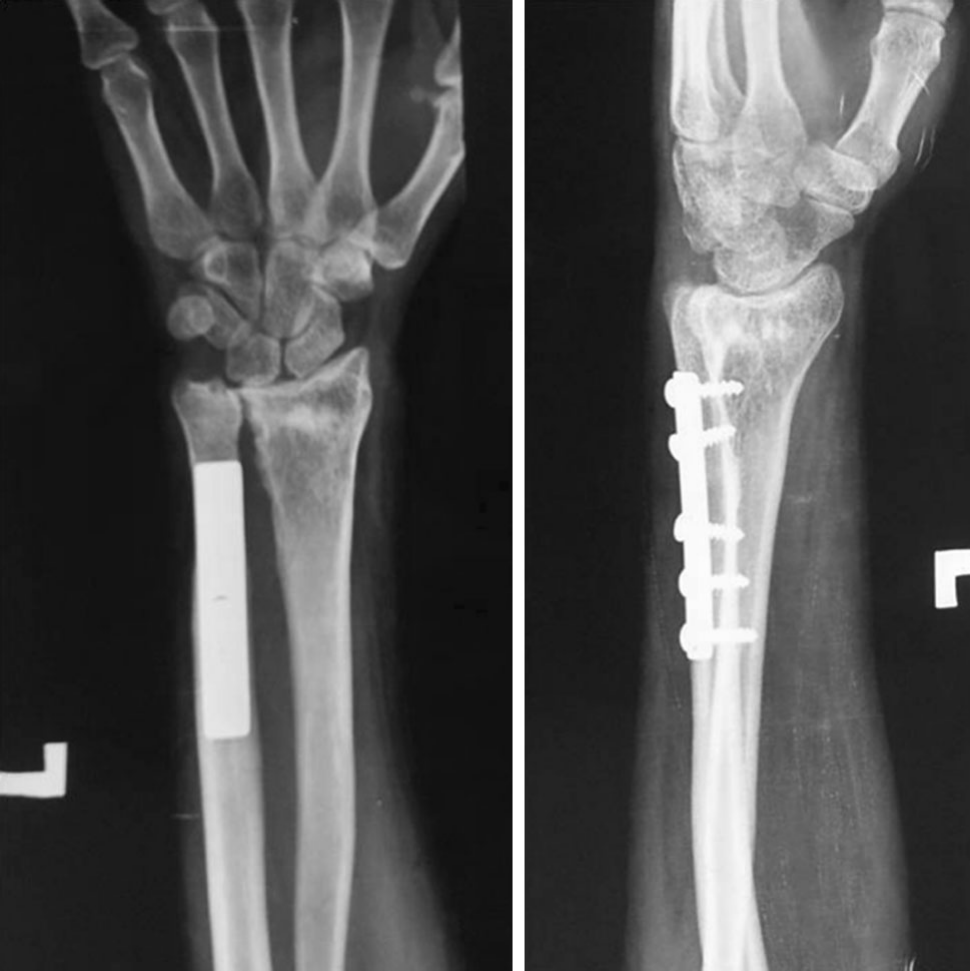
Radiographs at final follow-up at 2 years

The surgery was performed under tourniquet control. An 8-cm longitudinal dorsal midline incision was made on the distal forearm. To begin with, an ulnar shortening osteotomy was performed, taking care that a negative ulnar variance of 1–2 mm was attained. The osteotomy site was kept as distal as possible to minimize the risk of non-union. The distal end of the ulna was then pushed proximally and fixed using a plate at the proximal end. Using the same incision, a dorsal opening wedge osteotomy was performed on the distal radius in the region of the metaphysis, which was hinged volarly. The osteotomy site was grafted using the cortical grafts harvested from the excised ulna. The graft was filled dorsolaterally to restore the volar angulation and radial inclination to within the acceptable limits (volar tilt—neutral to 20° volar, radial inclination—to within 15° of the normal side). The radial construct was then stabilized with two Kirchner (K) wires, inserted from the radial styloid. The DRUJ was reduced and fixed with a partially threaded 4-mm cancellous screw inserted from the ulna to the radius in a tricortical manner. An above-elbow cast was applied postoperatively. Active finger movements were encouraged immediately after the surgery, along with limb elevation. Postoperative radiographs revealed adequate restoration of distal radius anatomy (Fig. 2). At 6 weeks the cast, K wires and the DRUJ screw were removed under conscious sedation and analgesia on an OPD basis, and wrist range of motion exercises were initiated (Fig. 3). Patients were followed up monthly for 6 months and thereafter at 3-monthly intervals. At the time of final follow-up (mean 30 months; range 24 to 36 months), radiographs showed healing of the osteotomy and maintenance of radiological parameters (Fig. 4).

## Results

**Fig. 5:**
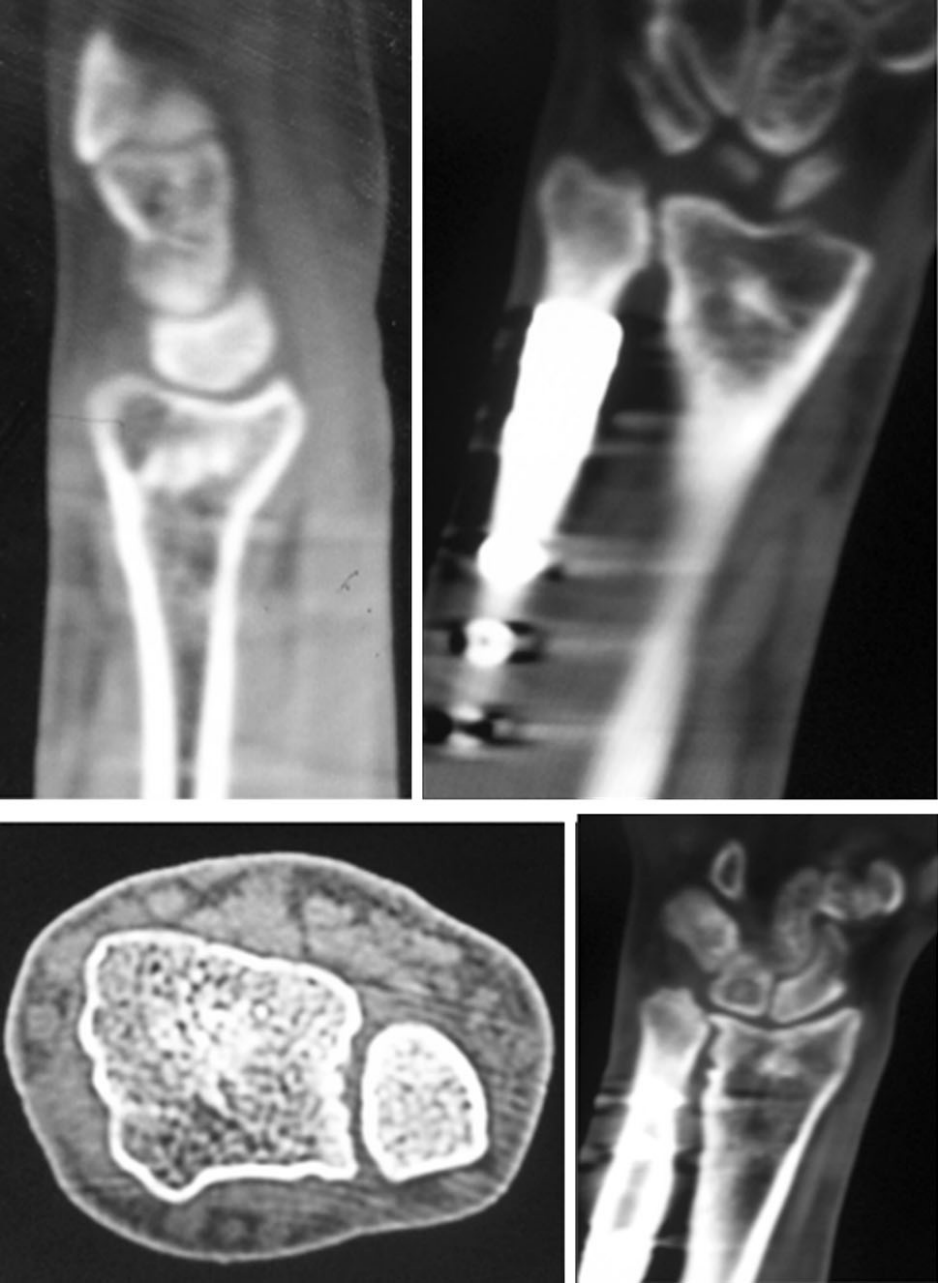
CT scan of wrist of same patient at 2 years

All patients got rid of the unsightly deformity and ulnar-sided wrist pain. Six months following surgery, patients noted marked improvement in flexion and rotations (Table 1). Mean dorsiflexion and palmar flexion increased by 10.8° and 30.8°, respectively. The increase in dorsiflexion was statistically highly significant (*p* = 0.0062), as was the increase in palmarflexion (*p* < 0.0001). Mean supination and pronation increased by 35.83° and 30.70°, respectively. The increase in supination was highly significant (*p* = 0.0006), as was the increase in pronation (*p* < 0.0001). Total arc of flexion increased from a mean of 60° to 102.5°. Total arc of rotation increased from a mean of 62.5° to 129°. The mean preoperative DASH score was 87.50, which improved to 18.73 postoperatively (*p* < 0.0001), which was highly significant. As the distal ulna had been dislocated for many years, the sigmoid notch of the distal radius may not be able to accommodate the distal ulna congruently. Hence reduction of the DRUJ after such a long time may cause incongruity of the joint and subsequent osteoarthritis. Also, the possibility of arthritis arising due to temporary arthrodesis cannot be ruled out. We performed a CT scan of the wrist at the time of final follow-up (Fig. 5) which showed a congruent sigmoid notch, but evidence of arthrosis was present in four out of six cases, although none complained of pain. The presence of arthrosis was not surprising given the duration of neglect and severity of deformities we were dealing with. All patients were satisfied with the outcome. Also, at final follow-up the radiological parameters were well maintained and DASH score improved in all patients (Table 2). During surgery, a negative ulnar variance of 1–2 mm was achieved in all cases (see Figs. 2 and 3 showing 2 mm of negative ulnar variance), but in due course of time four cases maintained a negative ulnar variance (−1 mm), while two cases had neutral variance (see Fig. 4). This may be due to some collapse at the radial osteotomy site.

**Table 1:** Clinical details of patients

S. no.	Age/sex	Age at time of injury (years)	Pre-op flexion (DF/PF) (degrees)	Post-op flexion (DF/PF) (degrees)	Pre-op rotation (supination/pronation) (degrees)	Post-op rotation (supination/pronation) (degrees)	Final follow-up (months)
1	21/M	11	30/40	40/70	45/25	70/60	24
2	24/M	10	25/40	30/65	30/30	65/60	36
3	22/F	12	25/35	30/75	40/30	70/70	26
4	21/M	12	20/30	35/65	20/30	70/60	28
5	22/M	9	25/40	45/70	35/40	60/60	30
6	23/F	10	20/30	30/60	20/30	70/60	36

*DF* dorsiflexion, *PF* palmarflexion

**Table 2:** Ulnar variance and DASH scores (pre-op and post-op at final follow-up)

S. no.	Pre-op ulnar variance (mm)	Post-op ulnar variance (mm) (final follow-up)	Pre-op DASH score	Post-op DASH score (final follow-up)
1	+9	0	80	15.8
2	+10	−1	93	20.8
3	+8	0	82	19.2
4	+10	−1	90	17.5
5	+9	−1	87	15.8
6	+11	−1	93	23.3

## Discussion

Malunion of the distal radius can result in biomechanical abnormalities in the radioulnar, the radiocarpal and the midcarpal joints [15, 16]. In the normal wrist, ~82 % of the axial load is distributed onto the radius, with the remaining 18 % being borne by the distal ulna through the triangular fibrocartilage complex (TFCC). With 2.5-mm radial shortening, this relationship changes so that the ulna bears 42 % of the axial load [17]. Continued shortening further increases ulnar load bearing and can result in symptoms of ulnocarpal impingement. Radial shortening has further deleterious effects in that it alters the congruency of the DRUJ and increases tension on the triangular fibrocartilage complex; these changes can result in increased pain and decreased rotation at the DRUJ, with nearly 50 % loss in pronation and ~30 % loss in supination with 10 mm shortening. Besides causing restricted range of motion around the wrist, fractures maluniting with residual dorsal angulation and DRUJ disruption also cause an unsightly deformity [4].

Most of the epiphyseal injuries of distal radius in children are Salter–Harris type 1 or 2, and they are commonly dorsally angulated [18, 19]. As compared to fractures of the mid shaft, the fractures of the distal forearm possess a greater remodeling potential [6], attributable to the fact that the distal growth plate of the radius accounts for 75 % of the bone’s length [20] which permits a substantial potential for remodeling. Age and distance from the growth plate have also been found to be important factors for the remodeling of forearm fractures in children. The potential for remodeling is maximal when the plane of deformity lies in the plane of motion of the adjacent joint [5]. Larsen et al. examined 70 fractures of the distal forearm in children with an angulation up to 28° and found that children under 10 years possess the ability to correct angulation up to 28°, but the potential for correction is decreased with greater angulation and age over 10 years [21]. Therefore, most investigators recommend that correction of angular deformities should be performed in children over 10–12 years of age [6, 21–23]. As all our cases were in age range of 9–12 years at time of injury, all had significant growth potential remaining. Malunion in these cases, together with relative lengthening of ulna lead us to retrospectively diagnose physeal growth arrest in these cases.

Deformity in all our cases was a combination of wrist extension (due to malunion in extension) and radial deviation (due to ulnar overgrowth), although the pattern of deformity may be variable depending on the site and extent of physeal arrest. Variable loss of radial inclination was present in all cases in the coronal plane. The radial deviation was noted only several years after the injury.

Volar plates have been used with success to treat malunions of the distal radius by combining them with a corrective osteotomy [24]. The advantage of a volar plate is that it does not require cast immobilization. The dorsal defect that is created after the opening wedge osteotomy requires filling with an appropriate bone graft. The graft may be packed in via the volar exposure; however, a limited dorsal approach is indicated to improve visualization. A volar approach not only involves thorough surgical dissection but also necessitates a separate incision for addressing ulnar shortening [4]. For dorsally angulated fractures, techniques involving a dorsal approach and fixation are known to improve radiological parameters, as well as pain and function [4, 24]. Wieland, Dekkers and Brink reported good results in their series of malunited distal radius fractures using a dorsal open wedge osteotomy with a dorsal plate without bone graft [25]. However, a prominent dorsal implant, extensor tenosynovitis, and rupture of extensor tendons have been reported as complications after use of a dorsal plate. Moreover, dorsally placed implants have thicker plates, raised screw heads, and they lack the ability to contour the plate to fit the bone [26, 27]. Though the advent of low-profile dorsal plates has solved this concern to some extent, this technique often requires dissection of the extensor retinaculum, and sometimes resection of Lister’s tubercle [27, 28].

The current technique retains the advantages of the dorsal approach, namely excellent exposure of the radius and ulna and minimal surgical dissection, and by using Kirschner wires instead of plate, the complications associated with dorsal plating are ameliorated. There is no need for a formal second surgery for implant removal, as K-wires and DRUJ screws were removed in an OPD setting. The excised ulna is used as a graft, further mitigating the morbidity associated with graft harvesting. The biggest advantage of the technique is that all the four components of the surgery, which include osteotomy of the distal radius, osteotomy of the ulna, harvesting the bone grafts and DRUJ reduction can be done through a single incision and in a single sitting. Also, cost of surgery is minimal, as we did not use volar locking plates. Though this technique requires cast immobilization, with aggressive physiotherapy good range of motion is gained.

Our recommendation is to utilize this technique for addressing neglected epiphyseal injuries leading to dorsal angulation of the distal radius, with positive ulnar variance and DRUJ disruption, as it leads to optimal outcome and minimal morbidity. However, studies with larger sample size and longer follow-up are required to further support this observation.
